# Light pollution alters the phenology of dawn and dusk singing in common European songbirds

**DOI:** 10.1098/rstb.2014.0126

**Published:** 2015-05-05

**Authors:** Arnaud Da Silva, Mihai Valcu, Bart Kempenaers

**Affiliations:** Department of Behavioural Ecology and Evolutionary Genetics, Max Planck Institute for Ornithology, Eberhard-Gwinner-Strasse, 82319 Seewiesen, Germany

**Keywords:** artificial night lighting, seasonality, song production, dawn chorus, dusk chorus, weather

## Abstract

Artificial night lighting is expanding globally, but its ecological consequences remain little understood. Animals often use changes in day length as a cue to time seasonal behaviour. Artificial night lighting may influence the perception of day length, and may thus affect both circadian and circannual rhythms. Over a 3.5 month period, from winter to breeding, we recorded daily singing activity of six common songbird species in 12 woodland sites, half of which were affected by street lighting. We previously reported on analyses suggesting that artificial night lighting affects the daily timing of singing in five species. The main aim of this study was to investigate whether the presence of artificial night lighting is also associated with the seasonal occurrence of dawn and dusk singing. We found that in four species dawn and dusk singing developed earlier in the year at sites exposed to light pollution. We also examined the effects of weather conditions and found that rain and low temperatures negatively affected the occurrence of dawn and dusk singing. Our results support the hypothesis that artificial night lighting alters natural seasonal rhythms, independently of other effects of urbanization. The fitness consequences of the observed changes in seasonal timing of behaviour remain unknown.

## 1. Introduction

Urbanization proliferates worldwide at an unprecedented pace [1]. Human activities related to urbanization lead to severe environmental changes, including habitat destruction, increasing local temperatures (‘heat-islands’ [2]) and chemical, noise and light pollution [3]. Light pollution, i.e. the use of artificial light at night, is expanding globally, with yearly growth rates of 6% [4], and is increasingly perceived as a problem for wildlife [5–7]. For example, artificial night lighting can lead to disorientation during sea-finding in marine turtles [8] or during migration in birds [9]. It has been estimated that millions of birds die each year by crashing into lighted structures [10]. However, the ecological and evolutionary consequences of artificial night lighting remain poorly understood. Recent studies have raised awareness that artificial night lighting can have other, more subtle effects on individuals, in particular, effects related to the modification of biological rhythms.

Several studies have implicated light pollution in changes in diurnal patterns of behaviour. In general, artificial night lighting causes diurnal animals to extend the period during which they are active, and may affect endogenous circadian rhythmicity [11]. For example, some diurnal songbird species forage at night in cities during winter [12], presumably facilitated by artificial night lighting. Several songbird species sing earlier around dawn and later around dusk, or even become nocturnal singers under the influence of artificial night light [13–17].

There is also evidence suggesting that artificial night lighting may modify the phenology of birds [18]. For example, urban common blackbirds *Turdus merula* breed up to one month earlier [19], and moult three weeks earlier compared with rural conspecifics, and these effects may be caused by light pollution [20]. In accordance with this, in blue tits, *Cyanistes caeruleus*, females exposed to street lighting started egg-laying on average 1.5 days earlier in the season than females breeding in dark territories in the same forest [14]. Such effects of light pollution on seasonal timing are expected, at least in temperate regions, because individuals use photoperiod as a proximate cue to determine the time of breeding [21,22]. Artificial night lighting may then interfere with this natural cue by modifying an individual's perception of day length, leading to changes in physiology and behaviour. For example, male common blackbirds exposed to light at night while kept indoors in individual cages showed earlier testicular growth and earlier peaks in circulating plasma testosterone [20]. Thus, we would expect that in temperate songbirds, males not only sing earlier in the morning or later in the evening, but also start producing dawn and dusk singing earlier in the season.

The primary aim of this study was to identify whether the occurrence of artificial night lighting is associated with changes in the phenology of the production of dawn and dusk singing in six common songbird species. In spring, during the breeding season, daily song production typically peaks before sunrise (dawn chorus) and—to a lesser extent—around sunset (dusk chorus, [23–25]). Dawn and dusk song are thought to function in the context of male–male competition (territory defence) and female choice [24,25], and variation in both the daily and seasonal timing of singing may thus have fitness consequences. We generally expect that males in lighted habitats will commence their dawn and dusk singing earlier in the season compared with conspecifics in dark habitats. Here, we compare the strength of this seasonal effect in six songbird species for which we previously assessed the effect on the daily timing of singing [14,17]. To this end, we recorded dawn and dusk song in 12 sites that varied in the presence of light (and noise) pollution. We specifically selected sites in non-urban areas, to reduce potential effects of confounding factors related to urbanization, such as increased temperatures or food availability, which may also affect phenology. The secondary aim of our study was to examine the effects of weather conditions (i.e. rain and relative temperature) on the occurrence of dawn and dusk singing throughout the season.

## 2. Methods

### (a) Study sites and data collection

Each day, between 6 January and 17 April 2012, we recorded all bird vocalizations around dawn and dusk in 12 forested sites (0.6–1.8 ha) in Southern Germany. Sites were chosen such that (i) half were affected by artificial night lighting (street lamps), (ii) within each light ‘treatment’, half were affected by traffic noise (vehicles driving on a busy road adjacent to the site), (iii) they were in non-urban areas and as similar as possible in parameters other than noise and light. Data from different sites can be considered to be independent, because sites were between 0.5 and 28 km apart and chosen to avoid clustering of the same light conditions. Two pairs of one lighted and one unlighted site were selected along the same road, allowing direct comparison under similar noise conditions. For a detailed description of the sites (one site had to be excluded from most analyses) and their locations, see [17].

At each site, we placed two Song Meter SM2+ (Wildlife Acoustics, Concord, MA; http://www.wildlifeacoustics.com/products/song-meter-sm2-birds) recorders on the ground, 70–130 m apart, in order to maximize song detection. We programmed each device to record sounds (stereo, sampling rate 22 050 per second) between 1.5 h before local sunrise until 1.5 h after local sunset (times based on the coordinates of each plot). Sound files were stored as wav files onto Secure Digital High Capacity cards (Laxer, Fremont, CA).

Each recording device also contained a temperature sensor (inside the box), which we programmed to log air temperature every 5 min. Temperature data were stored as text files onto the same digital data cards.

### (b) Data extraction

We analysed each recording using Song Scope v. 4.1.1 (Wildlife Acoustics, Concord, MA; http://www.wildlifeacoustics.com/products/song-scope-overview), as explained in detail in [17]. We noted the song of the six most common species at the study sites: European robin *Erithacus rubecula*, common blackbird, song thrush *Turdus philomelos*, great tit *Parus major*, blue tit and common chaffinch *Fringilla coelebs*.

On each day between 6 January and 17 April 2012, we noted for each species and at each recorder, whether dawn or dusk song was produced (yes/no). This was the case whenever we detected at least three song repetitions (strophes) within less than 5 min during the relevant period. The dawn chorus was broadly defined as singing that occurred in the period between 1.5 h before sunrise until 1.5 h after sunrise. The dawn chorus usually started before sunrise. Mean onset of singing (±s.d.) in min from sunrise: robin: −57.7 ± 19.1 (*n* = 686 recording days), blackbird: −53.2 ± 17.3 (*n* = 869), song thrush: −49.1 ± 14.8 (*n* = 522), great tit: −31.4 ± 25.6 (*n* = 1018), blue tit: −16.2 ± 25.3 (*n* = 874), chaffinch: −9.1 ± 18.0 (*n* = 766). The dusk chorus was broadly defined as singing that occurred in the period between 1.5 h before sunset until 1.5 h after sunset. The dusk chorus usually stopped before sunset for the great tit, the blue tit and the chaffinch, and after sunset for the robin, the song thrush and the blackbird. Mean cessation of singing (±s.d.) in minutes from sunset: robin: 29.8 ± 20.6 (*n* = 515), song thrush: 27.5 ± 11.3 (*n* = 509), blackbird: 19.3 ± 14.1 (*n* = 817), great tit: −16.0 ± 18.8 (*n* = 931), blue tit: −20.4 ± 23.7 (*n* = 737), chaffinch: −26.0 ± 22.0 (*n* = 462). In total, we analysed 1579 recorder days for dawn singing (i.e. the sum of the number of days analysed for each recorder, equivalent to 4737 h) and 1444 recorder days (4332 h) for dusk singing.

During each dawn and dusk period (as defined above) and for each recorder, we noted the presence of rain (yes/no; recognizable on the sonogram as broad-frequency, low-amplitude, continuous sound), and extracted temperature at sunrise/sunset from the text files. We excluded recordings when heavy rainfall made song detection unreliable (*n* = 6 days at dawn, *n* = 4 days at dusk).

### (c) Statistical analysis

All statistical analyses were performed with R v. 3.1.0 [26] and the R-package lme4 v. 1.1.7 [27]. We used generalized linear mixed models with binomial error distribution (fitted by the Laplace transformation), with ‘site’ and ‘recorder nested within site’ as random effects to control for variation owing to site and for similarity between the two recorders at each site. For each species and each period (dawn/dusk) separately, we tested whether the probability of singing was determined by the presence of artificial light (factor ‘light’: yes/no) in interaction with ‘date’, and by ‘rain’ (yes/no) and ‘relative temperature’. Because temperature and date were strongly correlated (Pearson correlations: dawn: *r* = 0.50, *n* = 87, *p* < 0.001; dusk: *r* = 0.66, *n* = 85, *p* < 0.001), we computed relative temperature as residuals of a mixed effect model with temperature (°C) as dependent variable, ‘date’ as fixed effect with random slope and ‘site’ as random intercept. For the song analyses, the intercept for the date was set to 1 January for the two tit species. For the other species, that started singing from mid-February onwards, the intercept was set at the median date for which they were singing in half of the sites (robin: 1 March, blackbird: 21 February, song thrush: 3 March, chaffinch: 23 February). We did not include traffic noise as a variable in the models, because (i) previous work suggested that traffic noise did not affect the daily timing of dawn and dusk singing [17] and (ii) there is no clear prediction about how traffic noise would affect the seasonal timing of dawn or dusk singing. We controlled for between-site variation in the presence or density of each species by removing those recorders where the focal species was singing during less than one quarter of the entire recording period from 15 February onwards (number of recorders excluded at dawn/dusk: robin 0/1, song thrush: 2/1 (absent from one lighted site), blue tit: 2/2, chaffinch: 1/5). We checked whether the song of the same individual was picked up by both recorders at a site. If this was the case (only one song thrush at each of two lighted sites), we removed the data from one recorder. In winter, malfunctioning of the recorders owing to battery problems led to missing data (January: 9 days at dawn, 12 at dusk; February: 6 days at dawn, 7 at dusk).

All tests are two-tailed, and *p*-values lower than 0.05 are considered significant. We report means and their standard errors.

## 3. Results

### (a) Natural variation in the phenology of dawn and dusk singing

**Figure 1.**
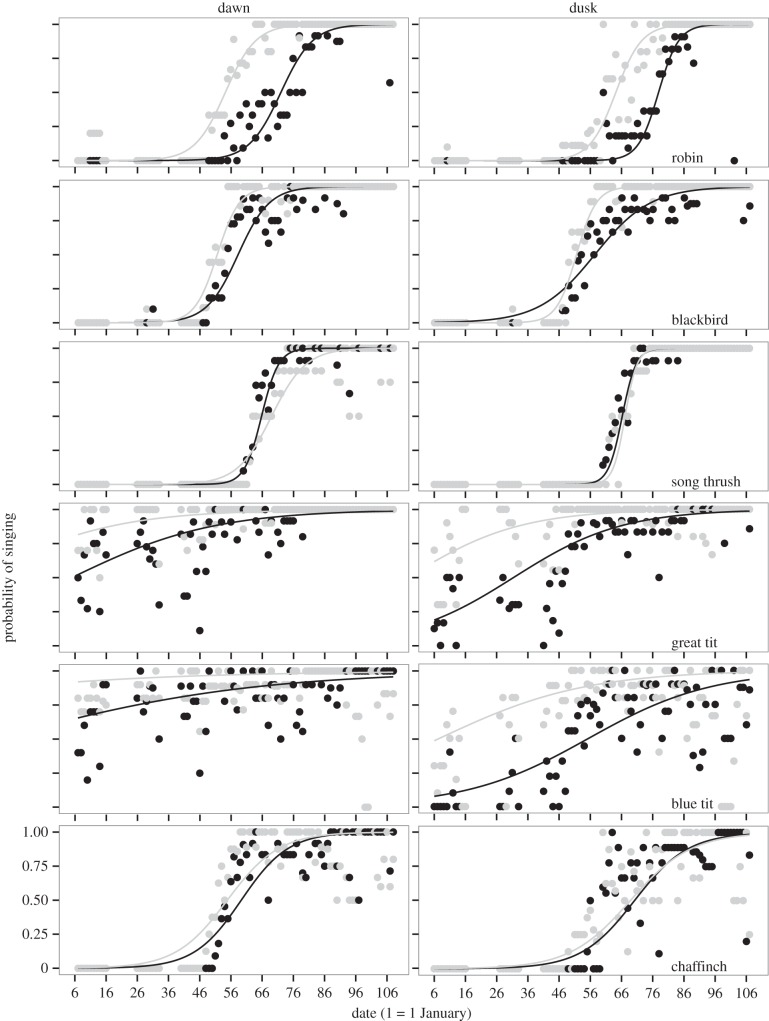
Observed and predicted daily probability of singing at dawn (left panels) and at dusk (right panels) for six songbird species at sites with artificial night lighting (grey dots and lines) and for non-lighted sites (black dots and lines). The dots show the proportion of recorders where dawn or dusk song was observed. Solid lines indicate means as predicted from generalized linear mixed models for each species at dawn (table 1) and at dusk (table 2), as described in §2c. Data are based on recordings from 6 January until 17 April 2012.

As expected, for all species, the probability of singing at dawn and at dusk increased from winter to breeding (main effect of date; figure 1 and tables 1 and 2). The two tit species had already started dawn and dusk singing when the recordings started, but the other species only started producing dawn or dusk song much later. Apart from isolated instances in early winter (five for robins, three for blackbirds, seven of them in the same lighted site), the earliest dawn chorus was recorded on 16 February (blackbird). For all species except the song thrush the dawn chorus seems to develop earlier in the season than the dusk chorus (figure 1).

**Table 1.** Effect of artificial night lighting, date and weather on the probability of singing at dawn. s.e., standard error.

predictors^a^	estimates	s.e.	Z	*p*
robin				
*intercept*	*−1*.*8*	*0*.*6*		
light^b^	+3.5	0.8	+4.2	<0.001
date	+0.2	0.02	+12.9	<0.001
date*light	+0.01	0.02	+0.4	0.7
rain^c^	−0.7	0.3	−2.7	0.007
temperature residuals	+0.1	0.04	+3.0	0.003
blackbird				
*intercept*	*−0*.*4*	*0*.*6*		
light^b^	+1.4	0.9	+1.6	0.1
date	+0.2	0.02	+12.4	<0.001
date*light	+0.1	0.03	+2.4	0.02
rain^c^	−1.4	0.3	−4.5	<0.001
temperature residuals	+0.3	0.05	+6.0	<0.001
song thrush				
*intercept*	*−1*.*0*	*0*.*6*		
light^b^	−0.07	0.8	−0.1	0.9
date	+0.4	0.04	+9.9	<0.001
date*light	−0.2	0.04	−4.5	<0.001
rain^c^	−0.4	0.3	−1.3	0.2
temperature residuals	−0.03	0.05	−0.6	0.6
great tit				
*intercept*	*+0*.*3*	*0*.*3*		
light^b^	+1.4	0.5	+2.6	0.009
date	+0.04	0.005	+9.0	<0.001
date*light	+0.002	0.01	+0.2	0.8
rain^c^	−1.4	0.2	−6.7	<0.001
temperature residuals	+0.1	0.02	+3.9	<0.001
blue tit				
*intercept*	*+0*.*6*	*0*.*7*		
light^b^	+1.8	1.0	+1.9	0.06
date	+0.02	0.004	+5.8	<0.001
date*light	−0.002	0.01	−0.3	0.7
rain^c^	−1.0	0.2	−5.0	<0.001
temperature residuals	−0.05	0.03	−2.0	0.04
chaffinch				
*intercept*	*−0*.*1*	*0*.*4*		
light^b^	+0.6	0.5	+1.1	0.3
date	+0.1	0.01	+13.9	<0.001
date*light	−0.01	0.01	−0.9	0.4
rain^c^	−1.3	0.2	−5.5	<0.001
temperature residuals	+0.2	0.03	+5.4	<0.001

^a^Variance explained by ‘site’ and by ‘site coupled with recorder’: robin: 1.7 and 0.0, blackbird: 1.3 and 1.0, song thrush: 1.5 and 0.1, great tit: 0.2 and 0.1, blue tit: 1.3 and 1.1, chaffinch: 0.5 and 0.4.

^b^Estimates are for lighted plots compared with non-lighted plots.

^c^Estimates are for rainy days compared with non-rainy days.

**Table 2.** Effect of artificial night lighting, date, and weather on the probability of singing at dusk. s.e., standard error.

predictors^a^	estimates	s.e.	*Z*	*p*
robin				
*intercept*	*−5*.*3*	*1*.*1*		
light^b^	+4.5	1.5	+3.0	0.003
date	+0.3	0.03	+9.9	<0.001
date*light	−0.1	0.04	−1.7	0.09
rain^c^	−0.9	0.3	−2.7	0.007
temperature residuals	+0.2	0.05	+3.4	<0.001
blackbird				
*intercept*	−*0*.*8*	*0*.*4*		
light^b^	+0.8	0.7	+1.2	0.2
date	+0.1	0.01	+11.2	<0.001
date*light	+0.2	0.04	+5.1	<0.001
rain^c^	−0.4	0.3	−1.4	0.2
temperature residuals	+0.1	0.04	+3.1	0.002
song thrush				
*intercept*	−*1*.*2*	*0*.*8*		
light^b^	−0.7	1.2	−0.6	0.6
date	+0.4	0.05	+8.6	<0.001
date*light	+0.1	0.1	+0.7	0.5
rain^c^	−1.1	0.5	−2.1	0.03
temperature residuals	+0.01	0.07	+0.2	0.9
great tit				
*intercept*	*−1*.*8*	*0*.*5*		
light^b^	+2.0	0.7	+2.6	0.009
date	+0.06	0.01	+11.4	<0.001
date*light	−0.002	0.01	−0.2	0.8
rain^c^	−1.2	0.2	−6.0	<0.001
temperature residuals	+0.1	0.02	+5.5	<0.001
blue tit				
*intercept*	−*2*.*6*	*0*.*6*		
light^b^	+2.5	0.8	+2.9	0.003
date	+0.05	0.005	+10.7	<0.001
date*light	−0.01	0.01	−1.2	0.2
rain^c^	−1.1	0.2	−6.2	<0.001
temperature residuals	+0.04	0.02	+1.9	0.06
chaffinch				
*intercept*	*−1*.*9*	*0*.*6*		
light^b^	+0.4	0.9	+0.4	0.7
date	+0.1	0.01	+10.0	<0.001
date*light	−0.01	0.01	−0.9	0.4
rain^c^	−1.8	0.3	−6.3	<0.001
temperature residuals	+0.2	0.03	+5.9	<0.001

^a^Variance explained by ‘site’ and by ‘site coupled with recorder’: robin: 4.9 and 0.6, blackbird: 0.9 and 0.3, song thrush: 2.5 and 0.3, great tit: 0.8 and 0.5, blue tit: 1.1 and 0.6, chaffinch: 1.8 and 0.0.

^b^Estimates are for lighted plots compared with non-lighted plots.

^c^Estimates are for rainy days compared with non-rainy days.

### (b) Effect of artificial night lighting on the phenology of dawn and dusk singing

Overall, the probability of dawn or dusk singing was affected by the presence of street lighting in five out of six species (interaction between light and date, or a main light effect; tables 1 and 2). The main effect of light reflects a difference in the probability of singing at dawn or dusk during the period when this probability is increasing, because all species are singing almost every day and at every site later in the recording period (figure 1).

Robins, blackbirds and great tits were more likely to produce a dawn chorus earlier in the season in the lighted sites compared with the dark sites (figure 1 and table 1). The blue tit showed a similar, but non-significant trend (figure 1 and table 1). The effect was in the opposite direction for song thrushes; they produced dawn song somewhat later in the season in the sites affected by artificial night lighting (figure 1 and table 1). In the chaffinch, the probability of dawn singing did not differ between lighted and dark sites across the entire recording period (figure 1 and table 1).

Similar to the dawn chorus, robins, blackbirds, great tits and blue tits were more likely to produce a dusk chorus earlier in the season in the lighted sites compared with the dark sites (figure 1 and table 2). In the song thrush and the chaffinch, the probability of dusk singing did not differ between lighted and dark sites across the season (figure 1 and table 2).

### (c) Effect of weather on the phenology of dawn and dusk singing

For all species, the probability of singing at dawn or dusk was reduced when it was raining, with the song thrush (not significant for dawn song) and the blackbird (not significant for dusk song) being the least affected (tables 1 and 2). Except for the song thrush and the blue tit, all species were more likely to produce a dawn chorus on days that were warm relative to the time of year (table 1), and a similar effect was detected for the dusk chorus (table 2).

## 4. Discussion

Our results show that artificial night lighting is associated with the phenology of singing in all the songbird species in this study, except the chaffinch. Male robins, blackbirds, great tits and blue tits (the latter only significantly at dusk) were more likely to sing earlier in the season at light-polluted sites, compared with conspecifics at non-lighted sites. Unexpectedly, the opposite effect was found for the song thrush: males were more likely to sing at dawn earlier in the season in the dark sites. We also found that the occurrence of dawn and dusk singing was weather-related: all species were less likely to sing when it was raining, and when it was relatively cold for the time of year.

### (a) The phenology of dawn and dusk singing

The six species under investigation showed a marked difference in their seasonal timing of singing (figure 1). Blue tits and great tits started dawn and dusk song earliest. The great tit was the most consistent in producing dawn and dusk singing over the entire period. In this species—and in the closely related blue tit—winter singing is more or less common, depending on the year [23]. Pairs can form or stay stable over winter, and males may use song to keep in touch with the partner [23]. Resident males may also use song to announce territory ownership inside winter flocks [28]. Resident chaffinches and blackbirds commenced morning and evening singing in mid-February (figure 1), on days of warm weather, and when food became more available [29]. They were followed later in February by the robin, which may be a partial migrant [30] in the area. Song thrushes started dawn and dusk singing the latest in the season (figure 1); this species rarely winters in Bavaria and most birds arrived in the study area in early March.

As the season progressed and breeding approached, the likelihood of dawn and dusk singing clearly increased (figure 1). This effect of date is mimicked by the daily timing of singing: all species started singing earlier relative to sunrise and (all except the blue and great tit) later relative to sunset closer to the start of breeding [17]. In the blue tit, this seasonal effect was also observed in sleep duration, even when controlling for differences in day length: males (and females) slept less as the season progressed [31].

Interestingly, dawn singing seems more dominant in early spring, with the dusk chorus becoming more prevalent later in the season, especially in the tit species, in line with Slagsvold [29]. The song thrush appears to invest more than the other species in the evening song peak, for reasons that remain to be studied.

In line with other work on song activity [17,29,32–35], we found that dawn and dusk singing was less likely on relatively cold or rainy days. Rain and colder temperatures may negatively impact song production because of the associated costs of singing in adverse conditions, or because other behaviours (e.g. foraging) become more important [33].

### (b) Effects of artificial night lighting on the phenology of dawn and dusk singing

At sites with artificial night lighting all species except the song thrush and the chaffinch were more likely to produce dawn and dusk song earlier in the season (figure 1). Thus, our study suggests that light pollution can lead to a faster seasonal development of the dawn and the dusk chorus. Interestingly, the effect was most pronounced in the robin and the blackbird (figure 1), which are the two species that naturally sing earliest at dawn and that are most affected by light in terms of earlier daily singing, whereas absent in the chaffinch, a species whose daily timing of singing was not affected by light [14,17]. The only exception is the song thrush, where artificial night lighting was associated with a (minor) delay in the development of dawn singing (figure 1), despite a similar effect on the daily timing of dawn song as in the robin and blackbird [17]. We discuss three non-mutually exclusive mechanisms that might explain the overall earlier annual initiation of dawn and dusk song peaks in sites with artificial light at night.

#### (i) Earlier seasonal growth of the brain song system owing to light pollution

In birds, a network of brain nuclei known as the song control system is responsible for song production and learning [36]. The size of these brain areas increases from the non-breeding to the breeding season through the creation of new synapses and new neurons [36–38]. Changes in the photoperiod are responsible for these changes in song nuclei volume [39,40]. In red-winged blackbirds (*Agelaius phoeniceus*), neurons from captive birds exposed to long (‘summer-like’) days have bigger dendritic fields than neurons from captive birds exposed to short (‘winter-like’) days [41]. The regulation of the song control nuclei and hence singing behaviour is influenced by hormones such as testosterone ([42–44] see also 4*b*(*ii*)), and melatonin [45–48]. The duration of melatonin synthesis in the pineal gland is proportional to the length of the night, and thus the melatonin cycle links changes in photoperiod to changes in the size of the song control nuclei. Melatonin thereby indirectly synchronizes the season with the singing behaviour of seasonally reproducing animals [18,48–50]. In late autumn and early winter, birds are already photosensitive [51]. Because light exposure suppresses melatonin secretion, artificial light at night reduces melatonin levels [18,52] such that short winter days may be perceived as longer spring days by the song control system. This may then cause males to sing earlier in the season provided weather conditions are clement.

#### (ii) Earlier seasonal development of the gonads owing to light pollution

As sexual hormones such as testosterone are also involved in the song control system [41–43,53], the activation of singing behaviour is directly linked to the photoperiodic activation of the reproductive system in birds. Indeed, the photoperiod is the main cue used by birds to synchronize their seasonal [22,54] and circannual rhythms [55], even for some tropical birds [56,57]. In temperate zone birds, the mean timing of gonadal growth and laying is proximately controlled by photoperiod [21]. Exposure to a succession of long days (or even to a single long day, see te Marvelde *et al*. [58]) causes adult birds to initiate gonadal growth. Exposure to artificial light at night thus has the potential to disrupt these patterns by stimulating sexual hormone secretion earlier in the season [18,59]. This may explain why blackbirds exposed to city illumination started reproducing three weeks earlier compared with blackbirds kept in the dark [20], and why female blue tits in territories with street lamps started laying a few days earlier compared with those in dark territories [14]. Thus, birds in lighted territories might already start the development of their reproductive organs in mid-winter, and may have higher levels of sex hormones than birds in the natural environment. Artificial night lighting may therefore shift the entire breeding phenology, provided there are relatively mild temperatures and sufficient food supply [60], causing earlier territorial aggression, mate guarding and dawn and dusk singing in males.

#### (iii) Increased residency owing to light pollution

Urban robins [30,61] and urban blackbirds [19,62] have a lower migratory disposition than rural conspecifics, probably because winter conditions are less severe in urban areas (higher temperatures and more food). Artificial night lighting may also play a role, for example if it allows longer foraging times in winter [12]. Individuals from woodland areas may also migrate into cities during winter rather than migrating further south [61]. Our lighted sites are not in urban areas, but they may still provide more food (or at least a longer period during which foraging is possible) than dark forest habitats during winter. Thus, robins and blackbirds may more often be found in winter in our lighted sites compared with dark control sites. We observed blackbirds at most sites during winter, irrespective of light presence. We did not observe any robins before mid-February and no song thrushes before early March, but we detected a few instances of nocturnal singing by a robin in January at a lighted site. Winter residency or earlier spring arrival may lead to earlier territory establishment and breeding, and hence to earlier singing, as we observed in robins and blackbirds. The song thrush initiated dawn singing later in the season in lighted sites, perhaps because it prefers breeding in less disturbed woodlands and only settled later in the lighted sites. Indeed, song thrushes seem more averse to human disturbance than blackbirds [63].

### (c) Conclusions and outlook

Our study suggests that artificial light at night advances the seasonal occurrence of singing in those songbird species that are also affected by light with respect to their daily timing of dawn and dusk singing (with the exception of the song thrush). Although we controlled for potentially confounding factors such as temperature, traffic noise and bird density (for more detail, see Da Silva *et al*. [17]), differences between lighted and non-lighted sites in, for example, quality of the males or arrival time could still have influenced our results. Experimental studies are now needed to confirm that the observed effects are indeed causally linked to the presence of artificial night lighting.

Our results add to accumulating evidence that light pollution has the potential to alter natural seasonal rhythms [18,20]. This and previous work on effects of light pollution suggest that birds breeding in lighted environments become territorial earlier and breed earlier than those in naturally dark habitats. However, the evolutionary consequences of the observed effects remain unknown. Singing earlier in the year could have positive fitness consequences for males, for example increasing the likelihood of attracting a (high-quality) social mate, or of siring extra-pair offspring [14]. Pairs could also compensate for smaller clutch sizes or lower productivity per nesting attempt in urban habitats by producing multiple broods owing to longer breeding seasons [64]. On the other hand, earlier singing or singing over a longer period may also come at a survival cost, owing to an increased risk of predation or because of exhaustion or elevated stress levels. Long-term individual-based studies are needed to address these issues, and to obtain a better understanding of the evolutionary consequences of artificial night lighting.

## Data accessibility

Data are available at http://behavioural-ecology.orn.mpg.de/repository/data/003/.
